# ERIRMS Evaluation of the Reliability of IoT-Aided Remote Monitoring Systems of Low-Voltage Overhead Transmission Lines

**DOI:** 10.3390/s24185970

**Published:** 2024-09-14

**Authors:** Halimjon Khujamatov, Dilmurod Davronbekov, Alisher Khayrullaev, Mirjamol Abdullaev, Mukhriddin Mukhiddinov, Jinsoo Cho

**Affiliations:** 1 Department of Computer Engineering, Gachon University, Seognam-daero, Sujeong-gu, Seongnam-si 1342, Republic of Korea; kh.khujamatov@gmail.com; 2 Department of Mobile Communication Technologies, Tashkent University of Information Technologies named after Muhammad al-Khwarizmi, Tashkent 100200, Uzbekistan; d.davronbekov@tuit.uz (D.D.); alisher02011993@gmail.com (A.K.); 3 Department of Information Systems and Technologies, Tashkent State University of Economics, Tashkent 100066, Uzbekistan; abdullaevm@tsue.uz; 4 Department of Communication and Digital Technologies, University of Management and Future Technologies, Tashkent 100208, Uzbekistan

**Keywords:** IoT, remote monitoring, low-voltage transmission lines, reliability, fault detection, overhead transmission

## Abstract

Researchers have studied instances of power line technical failures, the significant rise in the energy loss index in the line connecting the distribution transformer and consumer meters, and the inability to control unauthorized line connections. New, innovative, and scientific approaches are required to address these issues while enhancing the reliability and efficiency of electricity supply. This study evaluates the reliability of Internet of Things (IoT)-aided remote monitoring systems specifically designed for a low-voltage overhead transmission line. Many methods of analysis and comparison have been employed to examine the reliability of wireless sensor devices used in real-time remote monitoring. A reliability model was developed to evaluate the reliability of the monitoring system in various situations. Based on the developed models, it was found that the reliability indicators of the proposed monitoring system were 98% in 1 month. In addition, it has been proven that the reliability of the system remains high even when an optional sensor in the network fails. This study investigates various IoT technologies, their integration into monitoring systems, and their effectiveness in enhancing the reliability and efficiency of electrical transmission infrastructure. The analysis includes data from field deployments, case studies, and simulations to assess performance metrics, such as accuracy, latency, and fault detection capabilities.

## 1. Introduction

Monitoring events occurring during the production, transmission, and consumption of electricity is a priority for a country’s socioeconomic development. Recently, with the introduction of modern methods of corporate management, advanced information and communication technologies, and automated systems of energy accounting and control in the energy sector, increasing efficiency and reducing production costs, energy network organizations and financial institutions have played an important role in ensuring the transparency of companies’ activities [1]. Active efforts are being made to save energy, reduce energy consumption, and introduce advanced energy-saving technologies [2]. In particular, smart city (SC), smart energy (SE), and smart grid (SG) projects, which are organized based on the concept of the Internet of Things (IoT), are vivid examples. IoT is a system of interconnected physical objects, including devices, vehicles, and buildings, equipped with sensors, software, and other technologies to communicate and share data via the Internet. The IoT is transforming numerous industries by facilitating smarter decision-making, enhancing efficiency, and fostering the development of new business models [3].

Among the energy types, the design of electrical networks and systems, as well as the study of ways to increase their economic efficiency, reliability, and quality of electrical energy, are considered the basis for future promising projects [4].

It is known that, today, overhead and cable power lines are used to transmit electricity over long distances. Naturally, technical failures and interruptions occur in overhead power lines due to external influences and physical processes. A number of methods and tools are used to monitor these situations [5]. In most cases, the proposed methods provide monitoring of conditions such as vibrations in high-voltage overhead power lines, the vibration of transmission lines, lightning and strong wind effects, sag, magnetic radiation, and freezing of wires based on online monitoring using various sensors [6,7]. Existing electric field integral inversion methods have limited field conditions, and it is difficult to arrange electric field measurement points on high-span overhead lines. Research has proposed non-intrusive line voltage measurement by combining it with micro-sensor technology for power lines [8,9]. While high-voltage overhead power lines play a major role in transmission networks, low-voltage overhead power lines play an important role in the power transmission and distribution system.

Low-voltage overhead transmission power lines (OTPL) are crucial for distributing electricity from substations to end-users, including homes and businesses. Remote monitoring of these lines is very important because continuous monitoring ensures that the power supply remains stable and reliable, minimizing the risk of outages and disruptions. Furthermore, it identifies issues such as sagging lines and physical damage to prevent accidents and fires. Monitoring helps optimize the performance of the transmission network, reduce energy losses, and improve overall efficiency. Early evaluation of potential problems with the IoT allows for timely maintenance, reduces the likelihood of major failures, and extends the lifespan of the infrastructure [10,11,12].

New smart networks have emerged based on technological developments. Their monitoring requires the development of new methods and scientific solutions in the future [13,14,15]. Owing to the limitations of traditional low-voltage OTPL monitoring methods, such as being labor-intensive and time-consuming, difficulty in organizing monitoring in adverse weather and geographical areas, and providing only periodic data, the condition of power lines makes it difficult to obtain comprehensive information about their performance. Therefore, reliable IoT-based solutions are required to overcome these challenges. These solutions offer several advantages, such as continuous monitoring, comprehensive data collection, remote access, predictive maintenance, scalability, and flexibility, which address the limitations of traditional monitoring methods [16].

The main contributions of this study are as follows:

▪ This study aims to evaluate the reliability of an IoT wireless sensor network used for the remote status monitoring of low-voltage OTPL, and the proposed models provide a solid foundation for SG implementation.
▪ The supply sources of the sensor devices are perfectly configured, ensuring the delivery of information even in the event of a line failure.
▪ Reliable operation of the network is ensured by assessing the reliability of wireless sensors used in the remote monitoring of overhead power lines.
▪ Reliability assessment models and analytical expressions developed for remote monitoring systems and wireless sensor networks were compared with different methods and models and were found to be effective.
▪ On the basis of the information-graphic model, the possibility of the optimal use of the device is created.

Section 2 describes the related work, and the literature survey on the organization of the remote monitoring of overhead power lines based on IoT is reviewed. In Section 3, the research method and design parameters based on wireless sensor networks and IoT devices are proposed. In addition, the models and results of wireless sensor deployment in low-voltage transmission-line failure studies, remote monitoring systems, and wireless sensor deployment models and reliability were calculated and compared. Section 4 summarizes the conclusions and recommendations. Mathematical expressions and models developed to assess the reliability of the remote monitoring system of low-voltage overhead power lines based on IoT are presented. The results are compared with existing evaluation methods. Prospective projects expected within this research are discussed. 

## 2. Related Works

### 2.1. IoT-Based Remote Monitoring in Power Systems 

**Figure 1:**
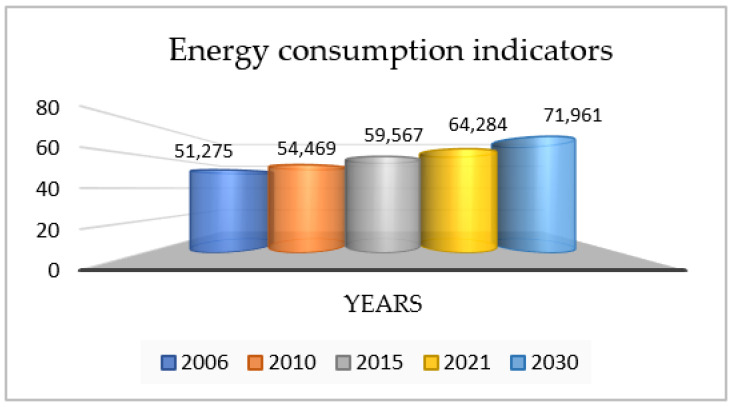
Indicators of energy consumption of global industrial sectors in 2006–2030 [17].

Current scientific research shows that due to Industry 4.0 (the Fourth Industrial Revolution), the energy consumption of the global industrial sector has increased significantly since 2006 (Figure 1). This situation has a significant impact on energy demand, which is forecast to reach 71,961,0 ZW by 2030 [17]. 

According to this analysis, most spent fuel is lost during the generation, transmission, distribution, and sale of electricity. Therefore, the problems and issues in this area have not been sufficiently researched.

It is possible to monitor the network remotely to reduce energy consumption in overhead power lines, detect technical faults, and prevent illegal connections. A SWOT analysis was performed to evaluate the advantages and disadvantages of the remote monitoring of overhead power lines (Table 1).

According to the results of the SWOT analysis, it can be seen that there are problems of interoperability with overhead power lines when implementing this method. A solution to this problem can be found in aspects such as the use of sensor devices in remote monitoring and the introduction of flexible and simpler models in the network by evaluating their reliability indicators [18].

The integration of the IoT in power systems has garnered significant attention owing to its potential to enhance monitoring and control mechanisms. Several studies have explored the application of IoT technologies in various aspects of power system management, including fault detection, energy management, and grid monitoring.

Several studies have been conducted worldwide to monitor the state of overhead power transmission lines. Zainuddin et al. discussed the conditions of overhead transmission lines in detail, including rising temperatures, voltage fluctuations, and how to overcome these conditions in their research [19]. They suggested that the materials used in cables in high-voltage overhead transmission lines should be composed of radiation- and heat-resistant elements. Their paper discussed the current state of controls to ensure that they can be used to detect overheating in OTPL conductors, as well as to monitor problems and obstacles. A few recommendations are provided in the conclusion of this study to address the incidence and analysis of temperature fluctuations. Finally, this research can provide comprehensive data for investigators and maintenance engineers, allowing them to make informed choices regarding system monitoring, operations, and maintenance. 

Based on the quality control methods, the factors affecting industrial interruptions (accidents) in overhead power lines must be investigated. Ishikawa and Pareto diagrams were proposed in [20], enabling them to compartmentalize the causal factors of technological violations. The authors’ publication outlines the primary accidental events and factors that have been analyzed and formalized that evaluate the reliability of overhead lines operation in distribution electrical networks with voltages ranging from 0.4–110 kV. The characteristics of the overhead lines of the AC power distribution electric grid that were considered are shown in Table 2.

Based on [20], the authors set the undertaking of decreasing the elevated level of collisions on overhead lines and enhancing the quality indicators of the electric power data services provided to consumers. To perform the specified tasks, a relevant data system for monitoring the specifications of the power lines was proposed.

### 2.2. Fault Detection and Monitoring 

In 2014, Christopher et al. proposed a distribution line monitoring system to detect electricity theft using a transmission line [21]. This system was primarily used to monitor the status of the transformers. This paper presents an innovative approach for identifying electricity theft based on the concept of power line communication. Because the standard measurement of high-frequency signals does not permit extremely complicated configurations, the implementation of this theft detection approach is highly recommended for power utilities. Therefore, this study has become the basis for the implementation of remote monitoring based on innovations to prevent future electricity theft.

Gungor and Hancke [22] discussed the use of industrial wireless sensor networks for condition monitoring in power systems, highlighting the role of IoT in real-time data acquisition and fault detection. Their study demonstrated that IoT-enabled sensors can provide continuous monitoring, allowing timely detection and localization of faults in transmission lines.

In [23], Ahmed and Kim focus on the implementation of IoT-based smart grid solutions for remote monitoring and fault detection. Their research emphasized the use of advanced sensor technologies and data analytics to enhance the reliability and efficiency of power distribution networks.

New approaches and limitations have been introduced for the transmission of information on power loss and line status. The existing technologies of data transmission networks and switching methods used in the transmission of status information on power lines were analyzed. Indian scientists conducted several studies on energy transmission losses. In particular, Dineshkumar and co-workers developed a GSM-based energy theft monitoring system centered on an embedded controller [24]. They presented a ubiquitous module for identifying electricity theft that can be assimilated into any type of energy meter. By adding a prudential meter, the module can be upgraded to detect illegal loads. This system assists governments in reducing electricity theft, thereby increasing revenue and grid power quality. However, this system does not provide the opportunity to monitor the condition of the line.

In overhead power lines, the energy loss or change in state is mathematically estimated by comparing the input and output powers in the network. In [25], energy loss with a mathematical analysis of electricity theft on the lines is presented as follows:

(1) $$\left\{ \begin{array}{l} \sum P_{sent}=P_{consued}+Loss\to No\cdot Thieft\\ \sum P_{sent}\neq P_{consued}+Loss\to Thieft \end{array}\right.$$

where, $P_{sent}$ is the amount of transmitted power and $P_{consumed}$ is the amount of power calculated by the consumer meter [25]. 

The development of novel wireless sensors for communication has led to their widespread use in various industries. The emergence of new generations of IoT has become the basis for the improvement of smart cities and grids. Particularly, they have been widely used to monitor the energy sector. In 2020, Muruganandhan et al. developed a model and software complex for the remote control of distribution transformers with the detection of power theft using Power Line Control (PLC) and supervisory control and data acquisition (SCADA) [26]. This software package is intended primarily for high voltage lines.

In addition, in this paper, the following research is comparatively analyzed (Table 3).

From the above analysis, it can be seen that the organization of remote monitoring systems for low-voltage overhead power lines based on IoT is simple, effective, and reliable. In particular, IoT-based evaluation of the reliability indicators of wireless sensors used to detect energy loss and technical faults in a network provides positive results. 

## 3. Proposal Methods

The main problems observed in OPTLs are technical failures and illegal connections to the line [34]. Under certain conditions, the network is turned off by the monitoring center, which is considered the main control point during line maintenance, and necessary measures are taken. In this case, the energy loss does not exceed the permissible amount [34,35,36]. However, in some cases, energy loss can be observed along the line owing to several factors. The illegal use of electricity is considered to have occurred in the following cases: when it is carried out without a consumer contract, when the metering equipment is bypassed, or when the meter is changed to change the indicators; the main goal is to save money.

### 3.1. Calculation Methods of Technological Losses in the Transmission and Distribution of Electric Energy through Electric Networks

It is important to consider the technical losses in the transmission and distribution of electrical energy through electrical networks. Technological losses: Technical losses of power transmission lines and power grid equipment spent on physical processes, losses associated with the allowable errors of the metering system of electrical energy transmitted between power supply enterprises, and the need for transformer substations are characterized by a value equal to the amount of electricity consumed. In this case, the accounting period is the period during which the norms of technological losses are considered (the next calendar year), and the base period is equal to the period before the accounting period (12 months). Technical losses consist of conditional fixed losses that do not depend on the transmitted power and load losses that depend on the transmitted power. Load loss ($∆W_{l.A.})$ during the transmission of electrical energy through regional power networks was determined using the following equation:

(2) $$\Delta W_{l.A}=\Delta W_{l.B.}\cdot \frac{W_{A.P.B}}{W_{A.P.A}}$$

where, $\Delta W_{l.B.}$ is the amount of electricity load loss in the base period, $W_{A.P.B}$ and $W_{A.P.A}$ are the amount of electricity provided (supplied) to regional power networks during the base and accounting periods, respectively. In this case, the electricity supplied to consumers through other electricity networks is not included in the volume. The volume of losses associated with the permissible errors of the accounting system during the transmission of electricity through regional power networks, $\left(\Delta W_{error.A}\right)$ is determined according to the following formula:

(3) $$\Delta W_{error.A}=\frac{\Delta W_{error.B\%}\cdot W_{A.P.B}}{100}$$

where, $\Delta W_{error.B\%}$ is the amount of electricity loss in the calculated base period due to permissible errors in the metering system (in percent).

### 3.2. Structure of Low-Voltage Overhead Power Transmission Networks

**Figure 2:**
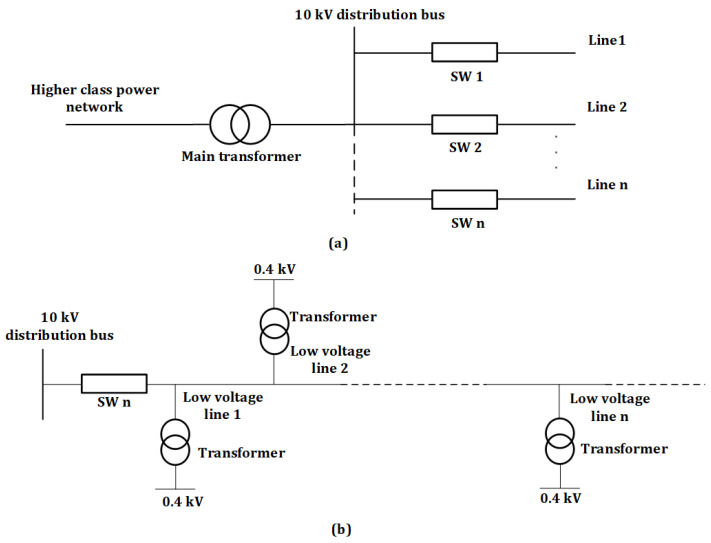
Model of the propagation process from the distribution transformer to the low-voltage line (**a**) transition from the high-power network to the distribution station; (**b**) transition from the distribution station to the low-voltage overhead transmission line [34,36].

As 0.4 kV overhead power lines were chosen as the object of research in this study, an analysis of the parts of the lines from the distribution point to the consumer was carried out (Figure 2).

Considering the large number of low-voltage distribution overhead power networks (0.38–6 kV), the number of transformer points of network sections can reach several hundred at the scale of the network economy [35]. Therefore, simple and inexpensive tools can be used to improve and change the voltage modes of electrical networks. Distribution networks with a voltage of 0.38–10 kV are described by multi-branching and long distances [34]. The schematic structure and operation of low-voltage distribution power networks are determined by the requirements (reliability) of their power supply, characteristics of consumers, and the industry networks to which they belong [36,37].

**Figure 3:**
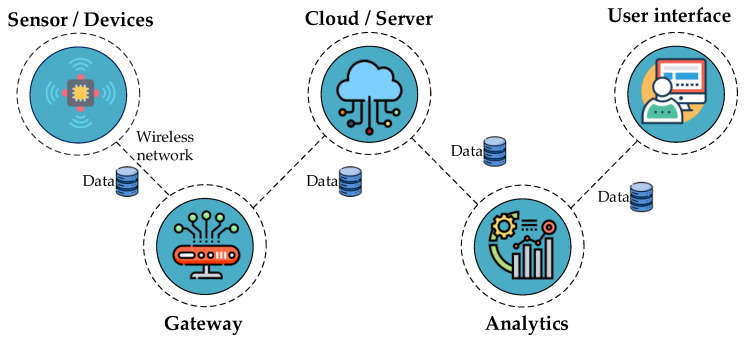
IoT Framework [37].

The use of IoT sensor devices has been proposed to perform these functional tasks more precisely and with better quality. The IoT is currently used in various fields. Using the IoT in a low-voltage OTPL can provide the desired results. In addition, it is necessary to consider the security aspects of hardware and connections. The main requirement for an IoT system is that this solution can be used by everyone, not only by specialists. The data obtained from the cloud system were analyzed to identify patterns and extrapolate knowledge [38]. Data visualization can be performed to make it easier for users to understand (Figure 3).

**Figure 4:**
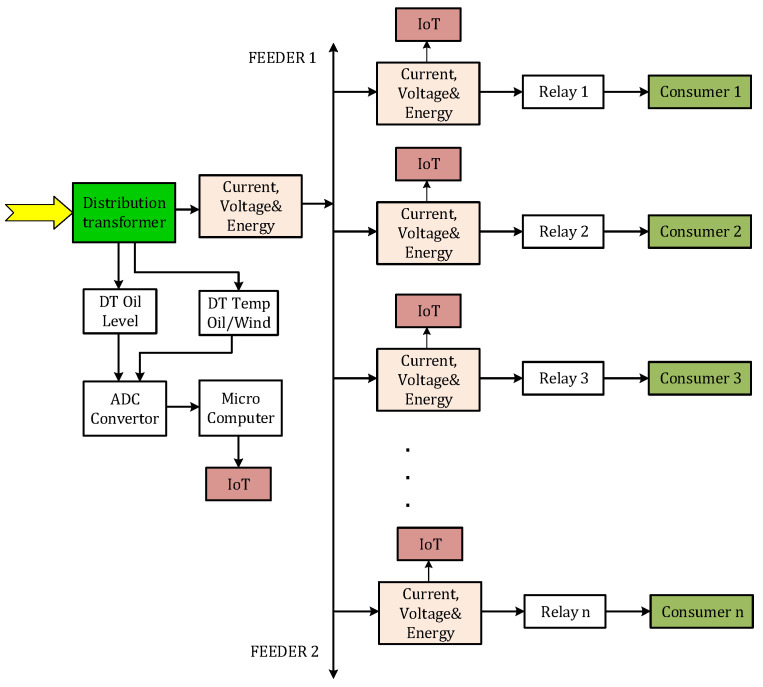
IoT deployment model between the distribution point and consumer meter.

The distribution transformer consists of a supply and a transmission part and distributes electricity to users on a 0.4 kV line. Figure 4 presents a model for using the IoT in automatic metering and energy control for the distribution point and user line. 

In this model, the voltage and current sensors (IoT sensors) monitor the secondary current, voltage, and amperage flows of the distribution transformer. The energy sensor controls the power sent from the distribution transformer. All sensors were connected to a microcomputer unit. This Wi-Fi-enabled microcomputer can be easily connected to personal computers using a web platform for remote monitoring of parameters.

### 3.3. Application of Wireless Sensor Networks in Remote Monitoring Systems

**Figure 5:**
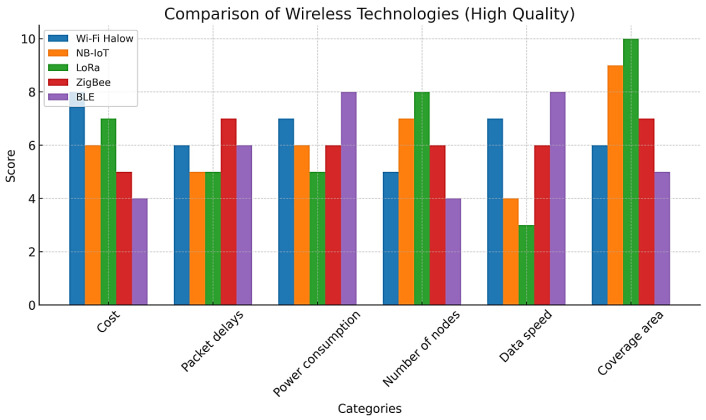
A comparison of different wireless network technologies.

The main goal of remote monitoring of the condition of overhead power lines based on wireless sensor networks is to measure and control the standard indicators set in each object using appropriate sensors, convert the monitored signals into digital form, and send them to the server of the monitoring center using wireless radio channels. One of the main issues in the organization of the monitoring system is the construction of a communication network on the scale of an overhead power line. The main problems that arise in this are the establishment of high-quality, reliable, and robust communication between the sensors affected by the electromagnetic field and the selection of wireless network technologies that can meet the necessary requirements. It was determined that ZigBee, Wi-Fi, BLE, NB-IoT, and LoRa technologies are used as wireless sensor technologies in the organization of remote monitoring systems [39], and these technologies are comparatively analyzed in Figure 5. 

The results of the comparison showed that there are mutual advantages and disadvantages in the characteristics of wireless technologies. For example, while LoRa dominates in terms of coverage area and the number of nodes in the network, ZigBee is preferable in terms of low cost and low energy consumption. Wi-Fi Halow, NB-IoT, and BLE technologies also have a number of advantages and disadvantages. But during the research, since the wireless sensor network for the remote monitoring system is being organized between electric mercuries (40–50 m between the mercuries), additional features of wireless technologies were also paid attention to, including retransmission, easy integration with existing systems, and resistance to electromagnetic field effects. According to the analysis, it was found that the use of ZigBee technology in remote monitoring systems of low-voltage overhead power lines is preferable and more effective than other wireless modules.

The system for operational monitoring of the technical condition of overhead power lines includes modular devices installed on the wires of low-voltage overhead lines, which transmit information through each other and are charged directly with the line. The topology of a sensor network depends on the tasks to be solved (the location of the sensors on the grid is affected by the task of diagnosing the parameters of the overhead power line and the requirement to retransmit the data). Communication channels are reserved in the system, which allows data transmission by bypassing the defective device not only within one phase wire, but also through devices on adjacent phase wires. The data collection module can be replaced by another monitoring task. The data received from the modular devices are collected in the permanent and additional memories of the concentrator.

**Figure 6:**
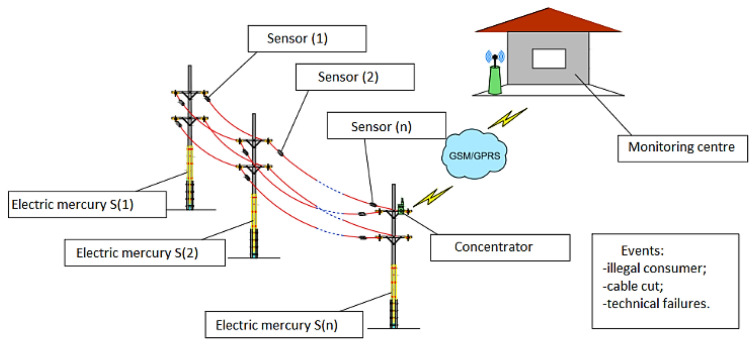
OTPL status remote monitoring structure deployment model between the distribution point and consumer meter.

The collected data are sent to the monitoring center using radio transmission modules and displayed on the computer of the monitoring center through a web interface (Figure 6).

**Figure 7:**
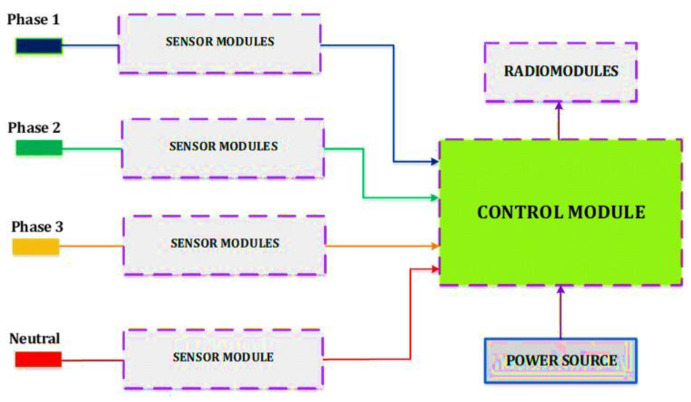
Remote monitoring device structure model.

The modular device serves as the basis for a network of sensors, consisting of a main board with a microcontroller and a communication module. The device was installed directly on a phase wire (Figure 7). The system consists of a sensor module, control module, diagnostic module, radio transmission module, and supply module. Sensor modules include voltage, current, and temperature sensors that collect information about the line condition and transmit it to a concentrator device. The devices installed on the transmission wires of the line are connected to the bus topology and send information to the next device via the ZigBee module.

The sensors are, respectively, voltage, current, and temperature sensors of the wires of the corresponding phase. They are separate devices containing primary measuring converters. They are functionally designed to generate a signal of measuring information, respectively, on the voltage, current, and temperature of the wire of one of the three electrical phases. In the particular case of the implementation of this device, the following sensors were used, respectively: ZMPT101b, SCT024T, and DS18B20. 

The microprocessor is performed using the Arduino MEGA platform, made on the ATmega2560 microcontroller, with a clock frequency of 16 MHz and 256 KB of flash memory for storing software. The A9G module is used as a data transmission and reception unit of the hub device, which combines a GSM/GPRS modem and a GPS/GNSS receiver in one device, which allows for organizing the transmission of wireless GSM/GPRS cellular data as well as tracking the location using GPS navigation. The ZigBee module of the TLSR8258 type is used as a data transmission and reception unit in the repeater device; the frequency range is 2.4–2.48 GHz, and the distance is 200 m.

The power supply element is a 3.7-volt battery that supplies DC power to all elements, units, and assemblies of the device. The device uses a 6800 milliampere-hour (mAh) lithium-ion battery. For elements, units, and assemblies that use different supply voltages, such as 5 V, a 3.7 V to 5 V converter of the LM2596 type can be used. All elements of the device, except for the sensors, are installed in a single housing made primarily of polystyrene, which stably isolates the device from external factors. Table 4 presents the errors during the monitoring tests of the sensors used in the developed device.

Wireless multicast communication is typically configured as a network with 20 or fewer terminals owing to complex routing. However, the total length of some long-distance OTPL is several tens of kilometers. Wireless networks with such power lines may not be able to transmit data over their entire length. In the proposed system, considering the installation of terminals along the line, it is possible to form a network by connecting up to 40 terminals to one concentrator, owing to the newly developed serial hopping mode. In this case, the main requirement for terminals in the system is a high reliability index of the device [37,38,40]. 

Several researchers have conducted studies to evaluate the reliability of monitoring systems. In particular, Golikov [41] classified reliability indicators based on analytical data according to different criteria depending on the specific conditions (constructive nature, technological nature, operational nature) of their use. The results of the work show the most important factors of reliability that can be determined quantitatively in heavy engineering using the direct expert evaluation method.

Jingde Huang and co-workers [42] conducted a study focusing on the limitations of traditional Bayesian networks in solving reliability problems of three-state systems, considering the classification, detection, and quantitative evaluation of uncertain data as the current problems in the field of high reliability system condition monitoring. The proposed three-state system reliability evaluation model by the authors helps to determine the real-time system reliability status, objectively reflects the actual operation process of the device, and allows early failure prediction.

In [43], Ivanovitch Silva and et al. discussed a methodology by which to assess the reliability of wireless sensor networks in a typical industrial environment. According to the proposed methodology, a WSN is modeled using a fault tree-based formalism, taking into account the problems occurring in the devices and the network.

Despite the conducted research, there are still limitations and obstacles in assessing the reliability of remote monitoring systems of overhead power lines based on wireless sensor networks. In this paper, reliability evaluation models of remote monitoring systems based on wireless sensor networks of low-voltage overhead power lines were proposed, and the results of the above models and the proposed model were compared. 

### 3.4. Reliability of Wireless Sensor Networks in Remote Monitoring Systems

Reliability is the property of maintaining the values of all parameters within the established time limits, which describes the ability of the system to perform the required functions in the given modes and conditions of use. Reliability is a complex property that includes four components: non-repudiation, reparability, durability, and maintainability [44]. Using a system made of sensors used in the OTPL as a non-renewable system, we evaluated their reliability indicators. Therefore, systems that cannot be recovered after irreversible rejection are unsuitable for further use. For some systems, non-recovery is defined by the terms of use where rejection cannot be corrected. Therefore, to solve the problems of estimating the reliability of the object and predicting its performance, it is necessary to have a mathematical model given by analytical expressions of one of the indicators *P*(t), f(t), or λ(t). The main way to obtain a model is to conduct tests, calculate statistical estimates, and approximate them using analytical functions. The non-rejection probability or reliability function *P*(t) represents the probability of non-rejection of a nonrenewable object up to the operation time t. This indicator has the following characteristics:

1. P(0)=1 (the object is introduced as having the ability to work until it is put into operation);
2. $\underset{t\to \infty }{\lim}P(t)=0$ (it is assumed that the object cannot maintain its working capacity indefinitely);
3. $\frac{dP\left(t\right)}{dt}\leq 0$, it is assumed that an object cannot be self-restored after a failure (this indicator is not used for systems that are recoverable by service personnel) [45].

**Figure 8:**
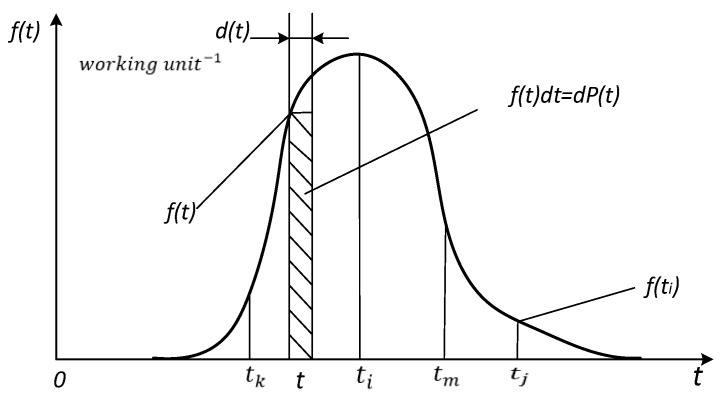
Density plot of the distribution of rejections [44,45].

The statistical definition of the rejection distribution density f(t) is as follows:

(4) $$\hat{f}(t)=\frac{\Delta n(t,t+\Delta t)}{N\cdot \Delta t},\left[{\mathrm{working}\;\mathrm{unit}}^{-1}\right]$$

where [t,t+t] is the operating interval, Δn is the number of rejected objects, *N* is the total number of objects, and *t* is the operation duration in Figure 8.

The statistical determination of the intensity of rejections *λ*(*t*) is described by the following expression [44]:

(5) $$\hat{\lambda }(t)=\frac{\Delta n(t,t+\Delta t)}{N(t)\cdot \Delta t},\left[{\mathrm{working}\;\mathrm{unit}}^{-1}\right],$$

That is, the intensity of rejection is the ratio of the number of rejected objects in the working interval [*t*, *t* + *t*] multiplied by the number of valid objects at moment *t* to the duration of the working interval *t*.

Many reliability assessment methods are currently being developed and used in practice. 

## 4. Result and Discussions

**Figure 9:**
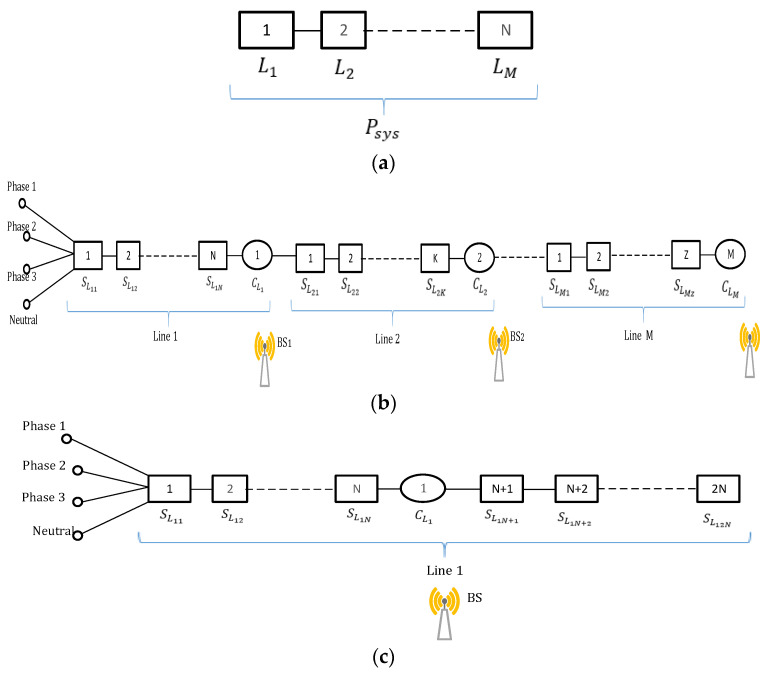
Reliability model of sensors in the network: (**a**) serial connection of sensors in the grid, (**b**) extended network model 1, (**c**) extended network model 2.

Since sensors and wireless modules are used in the main data transmission in the proposed system, this study uses the exponential distribution law to calculate the reliability of radio engineering systems to estimate the probability of line operation without rejection [46,47,48,49]. A network reliability model for sensors and concentrators in remote OTPL status monitoring is proposed in Figure 9 as follows:

In terms of calculations, the simplest case is the serial connection of the system elements. In these systems, the rejection of any element is equal to that of the entire system. Similar to a chain of conductors connected in series, the breaking of any one of them equals the breaking of the entire chain; we call such a connection a “series” connection [46,47]. We begin by estimating the successive reliability quantities. When evaluating system reliability, system elements are considered non-restorable objects. The evaluation was performed without considering the data transmission network of the system. Therefore, we expressed the reliability of the wireless network as follows: $P_{wcc}=1$. Given that all the sensors are made of the same elements and installed in the same manner, we calculated the probability of operation without rejection using the following expression:

(6) $$\left\{ \begin{array}{l} P_{L_{1}}=P_{S_{L_{11}}}\cdot P_{S_{L_{12}}}\cdot \ldots \cdot P_{S_{L_{1N}}}\cdot P_{C_{L_{1}}}\\ P_{L_{2}}=P_{S_{L_{21}}}\cdot P_{S_{L_{22}}}\cdot \ldots \cdot P_{S_{L_{1K}}}\cdot P_{C_{L_{2}}}\\ P_{L_{3}}=P_{S_{L_{31}}}\cdot P_{S_{L_{32}}}\cdot \ldots \cdot P_{S_{L_{3N}}}\cdot P_{C_{L_{3}}}\\ \vdots \\ P_{L_{M}}=P_{S_{L_{M1}}}\cdot P_{S_{L_{M2}}}\cdot \ldots \cdot P_{S_{L_{M_{Z}}}}\cdot P_{C_{L_{M}}} \end{array}\right.$$

It follows from Equation (5) that $P_{S}=P_{L_{1}}\cdot P_{L_{2}}\cdot \ldots \cdot P_{L_{M}}$ (6). If the following relation holds for all sensors:

(7) $$\left\{ \begin{array}{l} P_{S_{L_{11}}}=P_{S_{L_{12}}}=\ldots =P_{S_{L_{MZ}}}=P_{S_{Z}}\\ P_{C_{L_{1}}}=P_{C_{L_{2}}}=\ldots =P_{C_{L_{iM}}}=P_{C} \end{array}\right.$$

then the reliability probability of each isolated line is as follows:

(8) $$\left\{ \begin{array}{l} P_{L_{1}}=P_{C_{L_{1}}}\cdot \prod_{i=1}^{N}P_{S_{L_{1i}}}\\ P_{L_{2}}=P_{C_{L_{2}}}\cdot \prod_{i=1}^{K}P_{S_{L_{2i}}}\\ \vdots \\ P_{L_{M}}=P_{C_{L_{M}}}\cdot \prod_{i=1}^{Z}P_{S_{L_{Mi}}} \end{array}\right.$$

Hence, if we calculate the non-rejection probability for the system, we obtain a value equal to

(9) $$\begin{array}{l} P_{S}=P_{L_{1}}\cdot P_{L_{2}}\cdot \ldots \cdot P_{L_{M}}\\ P_{sys}=P_{C_{L_{1}}}\cdot P_{C_{L_{2}}}\cdot P_{C_{L_{M}}}\cdot \prod_{i=1}^{N}P_{S_{L_{1i}}}\cdot \prod_{i=1}^{K}P_{S_{L_{2i}}}\cdot \prod_{i=1}^{Z}P_{S_{L_{Mi}}} \end{array}$$

Considering that the system consists of sensors and concentrators, the general reliability function is expressed as

(10) $$\left\{ \begin{array}{l} P_{C_{L_{1}}}\cdot P_{C_{L_{2}}}\cdot \ldots \cdot P_{C_{L_{M}}}=P_{C}^{M}\\ P_{sys}=P_{C}^{M}\cdot P_{S}^{N+K+Z} \end{array}\right.$$

Considering Equation (10), the reliability of the system is characterized by an amount equal to the product of the reliability of the devices and their software:

$$P_{sys}=f(P_{S}),\;P_{sys}=f(P_{C})$$

(11) $$\left\{ \begin{array}{l} P_{sensor}=P_{hardware}\cdot P_{software}\\ P_{hardware}=e^{-\lambda_{hardware}\cdot t}\\ P_{software}=e^{-\lambda_{software}\cdot t}\\ P_{sensor}=e^{-(\lambda_{hardware}+\lambda_{software})\cdot t} \end{array}\right.$$

We obtain the general result as in Equation (11), where *λ(t)* represents the intensity of processes. Based on these expressions, the reliability indicators of the system were evaluated, and simulation modeling was performed using MATLAB and Proteus.

The following boundary conditions were included in the reliability calculation of the wireless sensor network used in the OTPL:

– connection of sensors and concentrators in a functional sequence;
– invariance of the intensity of rejections $\lambda \left(t\right)$ during operation (λ=const);
– that the elements in the system obey the exponential law [50,51].

P(t) and λ(t)*P*(t) of sensors and concentrators are equal to each other, that is:

(12) $$\left\{ \begin{array}{l} P_{S_{1}}=P_{S_{2}}=P_{S_{3}}=\ldots =P_{S_{N}}\\ P_{C_{1}}=P_{C_{2}}=P_{C_{3}}=\ldots =P_{C_{K}} \end{array}\right.$$

(13) $$\left\{ \begin{array}{l} \lambda_{S_{1}}=\lambda_{S_{2}}=\lambda_{S_{3}}=\ldots =\lambda_{S_{N}}\\ \lambda_{C_{1}}=\lambda_{C_{2}}=\lambda_{C_{3}}=\ldots =\lambda_{C_{K}} \end{array}\right.$$

If we analyze two different connection methods when evaluating the reliability of the system, the rejection intensity λ(t) of the system is expressed as follows:

(14) $$\lambda_{sys_{1}}=\lambda_{S_{11}}+\lambda_{S_{12}}+\ldots +\lambda_{S_{1N}}+\lambda_{C_{11}}+\lambda_{C_{12}}+\ldots +\lambda_{C_{1K}}$$

(15) $$\lambda_{sys_{2}}=\lambda_{S_{21}}+\lambda_{S_{22}}+\ldots +\lambda_{S_{2M}}+\lambda_{C_{21}}+\lambda_{C_{22}}+\ldots +\lambda_{C_{2L}}$$

(16) $$\lambda_{sys_{1}}=\sum_{i=1}^{N}\lambda_{S_{1i}}+\sum_{j=1}^{K}\lambda_{C_{1j}}$$

is for the first connection;

(17) $$\lambda_{sys_{2}}=\sum_{i=1}^{M}\lambda_{S_{2i}}+\sum_{j=1}^{L}\lambda_{C_{2j}}$$

This is the second connection. Given that P(t) obeys the exponential law, the probability of the system working without rejection in two different connections is

(18) $$P_{sys_{1}}=e^{-\lambda_{sys_{1}}\cdot t},\;P_{sys_{2}}=e^{-\lambda_{sys_{2}}\cdot t}$$

**Figure 10:**
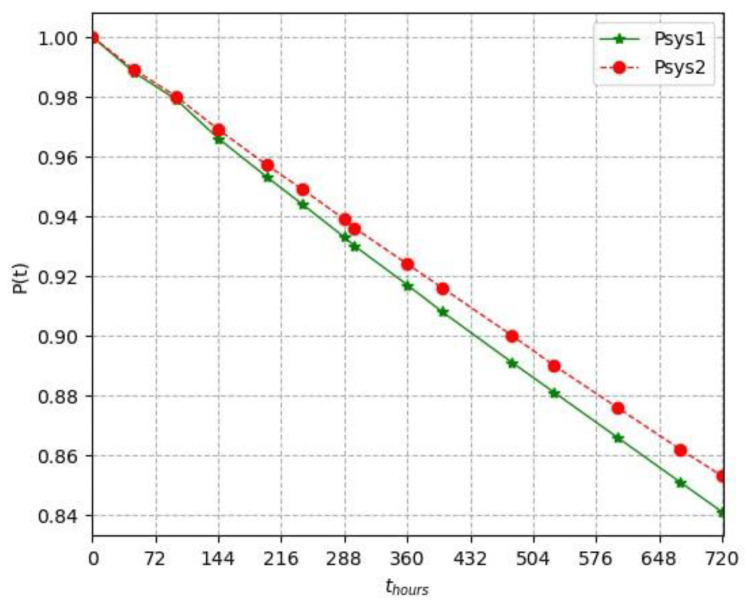
Graph of the probability of failure-free operation of lines in different connections.

To express the results of these analyses more precisely, the probability of the reliable operation of the system with two different connections during one month (720 h) is calculated (Table 5, Figure 10).
For the first grid N = 200 K = 10For the second grid N = 200 K = 5

$$\lambda_{S}=10^{-6}\frac{1}{hour},\;\lambda_{C}=4\cdot 10^{-6}\frac{1}{hour}$$

(19) $$\lambda_{sys_{2}}=200\cdot 10^{-6}\frac{1}{hour}+5\cdot 4\cdot 10^{-6}\frac{1}{hour}=220\cdot 10^{-6}\frac{1}{hour}$$

(20) $$\lambda_{sys_{2}}=200\cdot 10^{-6}\frac{1}{hour}+5\cdot 4\cdot 10^{-6}\frac{1}{hour}=220\cdot 10^{-6}\frac{1}{hour}$$

In this study, the reliability issues of the wireless sensor network used in the remote monitoring system of low-voltage overhead power lines were considered in two different extended models. From the analysis it was found that the second extended model had a higher reliability index than the first because the second model had fewer concentrators. 

**Figure 11:**
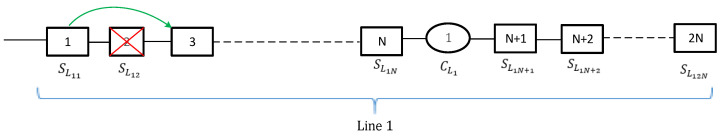
Network structure when one sensor fails, where the green line represents the transmission of information from sensor 1 to sensor 3, and the red line represents the failure (reject) of sensor 2.

We evaluate how the reliability of the network changes in the case of failure of the elements of the system in connection model 2 above. We express the assessment of the reliability of the sensor system more precisely; we introduce the following conditions (Figure 11):

– The maximum distance of information propagation was within the range of the three sensors in a sequence, and it was not transmitted over a greater distance.
– Only one sensor can fail (reject) on one line at the moment.

**Figure 12:**
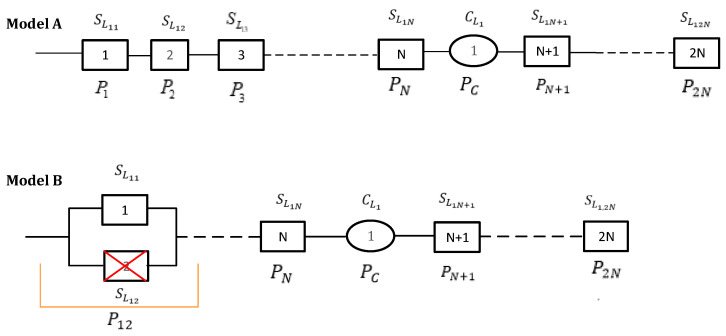
Reliability structural model of the system for different connection methods. The red line means that the sensor 2 has rejected, and the orange line means that the first and second sensors are connected in parallel due to the failure of the sensor 2.

In the case of the failure of one sensor in the system (Model B), we make a comparative comparison with the case of operation without the failure of all sensors in the initial connection (Model A) to represent the change of the reliability indicator of the system over time (Figure 12). In this case, the red line means that the sensor 2 has rejected, and the orange line means that the first and second sensors are connected in parallel due to the failure of the sensor 2.

For the models above, we calculated the probabilities of operation of the sequential system without rejection:

Model A. Given that the condition above is satisfied, the equation can be expressed as follows:

(21) $$P_{sys_{1}}=e^{-\lambda_{C}\cdot t}\cdot e^{-\lambda_{S}\cdot 2N\cdot t}=e^{-t\left(\lambda_{C}+\lambda_{S}\cdot 2N\right)}$$

Model B. In this model, sensors 1 and 2 are connected in parallel and are defined by the following:

(22) $$P_{1,2}=\left[1-\left(1-P_{S_{1}}\right)\cdot \left(1-P_{S_{2}}\right)\right]=2P_{s}-P_{S}^{2}$$

For Model B, we continue to calculate the probability of the non-repudiation of devices in the public network using $P_{sys_{2}}$

(23) $$\begin{array}{l} P_{sys_{2}}=\left(2P_{s}-P_{S}^{2}\right)\cdot P_{S_{3}}\cdot P_{S_{4}}\cdot \ldots \cdot P_{S_{2N}}\cdot P_{C}\\ P_{sys_{2}}=\left(2P_{s}-P_{S}^{2}\right)\cdot P_{C}\cdot \prod_{i=1}^{2N-2}\cdot P_{S_{i}} \end{array}$$

If we apply Equation (21), we obtain Equation (24)

(24) $$P_{sys_{2}}=\left(2e^{-\lambda_{S}\cdot t}-e^{-2\lambda_{S}\cdot t}\right)\cdot e^{-\lambda_{C}\cdot t}\cdot e^{-\left(2N-2\right)\cdot \lambda_{S}\cdot t}=\left(2e^{-\lambda_{S}\cdot t}-e^{-2\lambda_{S}\cdot t}\right)\cdot e^{-\left[\lambda_{C}+\left(2N-2\right)\cdot \lambda_{S}\right]\cdot t}$$

**Figure 13:**
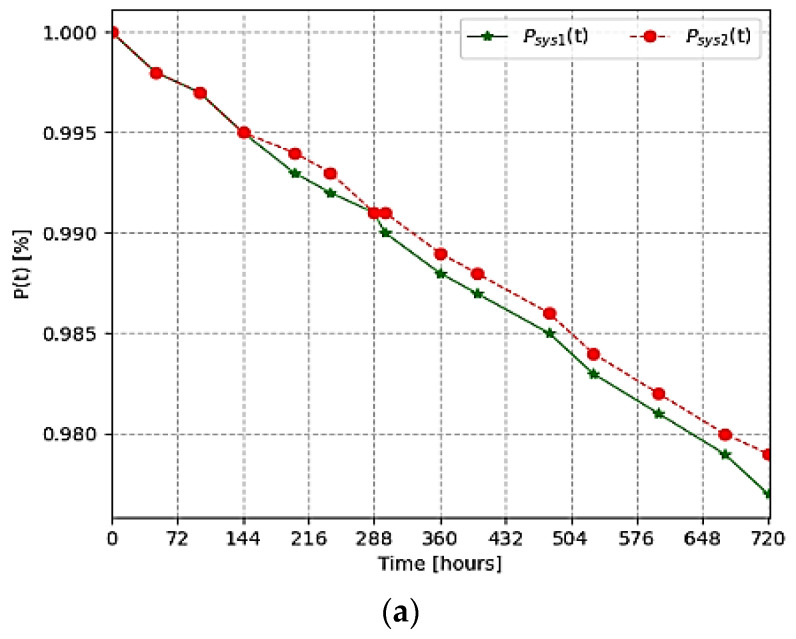

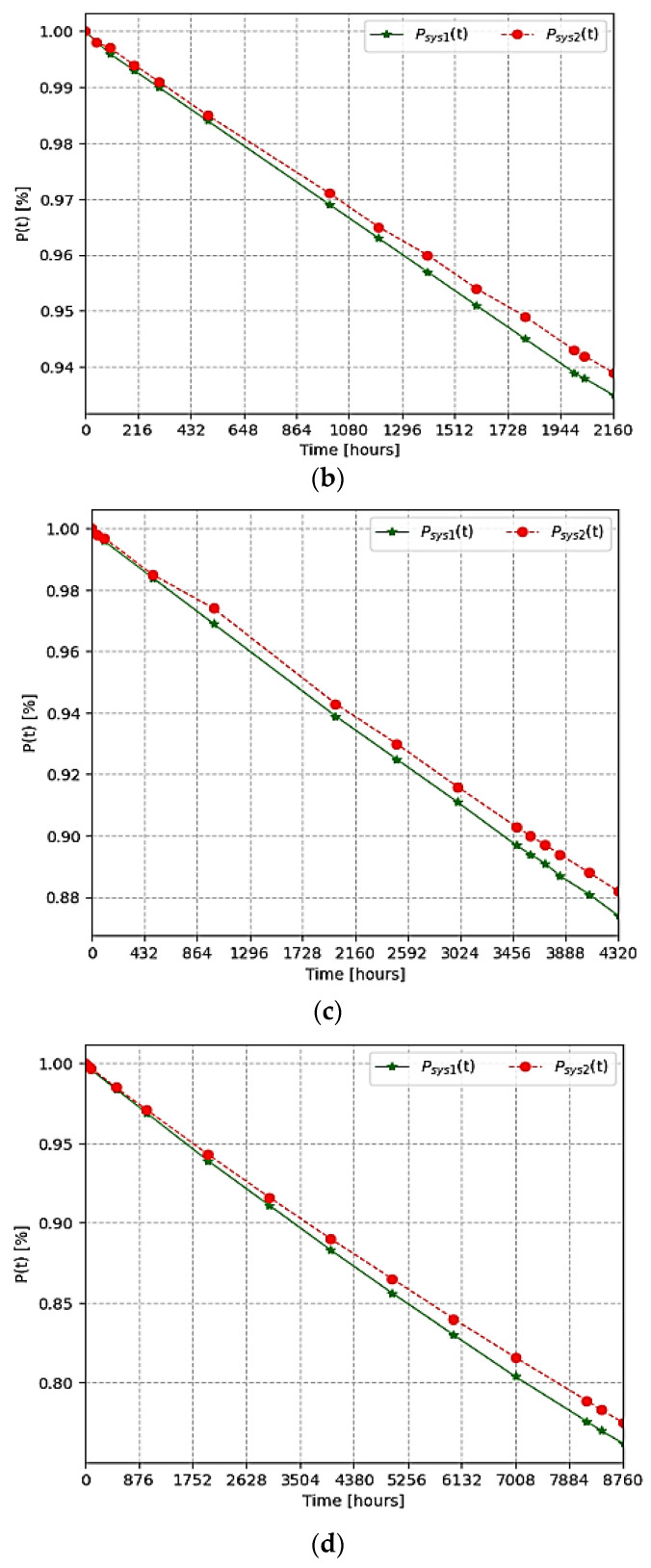
Reliability indicators of various models during a year: (**a**) for a month, (**b**) for three months, (**c**) for six months, (**d**) for a year.

Example 2. To express the results of these analyses more precisely, we calculated the probability of reliable operation of the system with two different connections over one year (8760 h) using Equations (21) and (24) in Table 6 and Figure 13. In this case, the values were assumed equal to

$$N=15,\;\lambda_{S}=\lambda_{C}=10^{-6}\frac{1}{hour}$$

**Figure 14:**
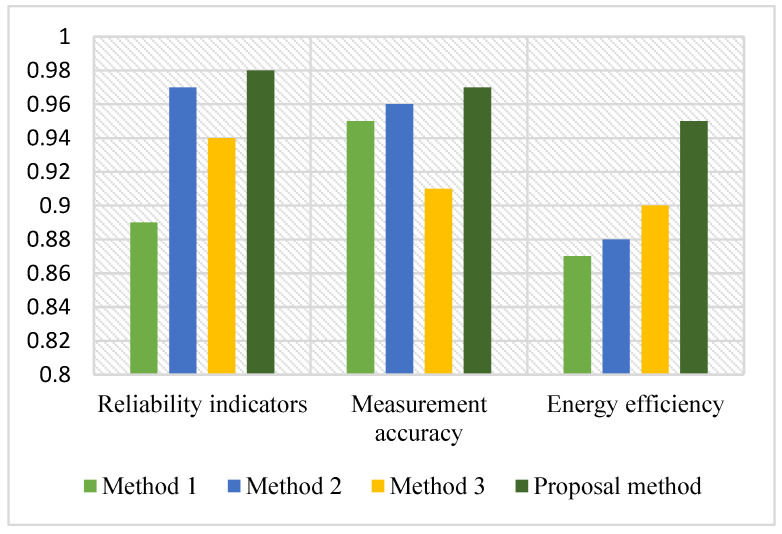
Comparison of the proposed model with the results of other researches.

During the research, the results of the system reliability assessment were analyzed in a month, 3 months, 6 months, and one year. From these equations and the results of the proposed models, it can be seen that even if one sensor in the sensor network of the remote monitoring system fails, the reliability index of the system is relatively high. The abovementioned models serve to estimate the reliability indicators of low-voltage overhead lines based on WSN. The results of the proposed model and the reliability assessment of the monitoring processes of different industries presented in [41,42,43] were compared. The results of the comparison are given in the following Table 7 and Figure 14.

It was found that the reliability evaluation model of a low-voltage overhead power line condition remote monitoring system is higher than other evaluation models. In addition, the reliability model of the remote monitoring system based on wireless sensor networks of low-voltage overhead power lines developed as a non-restorable system is one of the new innovative methods proposed in this field.

This study underscores significant progress in IoT technologies for the monitoring and management of power systems. Specifically, IoT-based remote monitoring systems for low-voltage overhead transmission lines provide greater reliability, efficiency, and fault detection compared to conventional approaches. These studies serve as a strong basis for the continued exploration and innovation of IoT solutions in this area. Utilizing IoT technologies for large-scale monitoring in modern intelligent networks is a key strategy for advancing the informatization of power grid systems, enhancing their stability, reliability, and efficiency. The growing need for automation and intelligent systems will drive the deep integration of IoT with smart grids (SG). Established wireless communication and network optimization theories form the theoretical basis for IoT applications.

In the remote monitoring of 0.4 kV overhead transmission power lines, sensors are selected based on the specific environment and conditions. For instance, if the temperature in the transmission line rises significantly, the chosen sensor must be able to withstand these temperatures. Considering the device’s energy consumption, low-power sensors, microcontrollers, and radio modules are preferred.

The remote monitoring of low-voltage overhead power lines using wireless sensor networks can encounter several obstacles, such as signal interference, limited battery capacity, external factors like weather, small network coverage areas, and outages. Additionally, data security and confidentiality concerns pose challenges to the effective implementation of the research method. Addressing these limitations through the integration of future artificial intelligence tools and innovations is a key focus of the research’s prospective projects.

## 5. Conclusions

The proposed models demonstrate that even if one sensor in the monitoring system fails, the overall system reliability remains high. In the two types of connections compared, Model B achieved a 1-month reliability index of 98%, which is 0.2% higher than Model A. Further assessments revealed that the reliability of Model B was 93% after 3 months and 88% after 6 months, making it 2–3% more reliable than Model A. If the system experiences more failures over time, redundancy measures are implemented to enhance the system’s reliability and efficiency. Additionally, it was found that the reliability evaluation model for the low-voltage overhead power line condition remote monitoring system, designed as a non-restorable system, is more reliable than models used in other monitoring fields.

A structural scheme and reliability model were developed to ensure the safe and reliable transfer of sensor data to the remote monitoring system. The system’s probability of functioning without failure and its rejection rate were calculated. To handle the large volume of sensor data, a microcontroller and additional memory were used. This research can be extended in new directions in the future.

## Figures and Tables

**Table 1:** SWOT analysis of the application in remote monitoring of overhead power lines.

**Strengths**	**Weaknesses**
-prevention of cyber-attacks on energy supply; -Smart modeling;	-using several network devices; -increase in expenditure; -the necessity of obtaining major investments for the advancement of technology
**Opportunities**	**Threats**
-the ability to remotely monitor the network in real time; -detection of technical malfunctions and illegal connections; -optimization of energy loss and assessing network reliability.	-disproportionate adaptation of network with sensor devices; -electromagnetic field effect; -improper organization of the system.

**Table 2:** Overhead line parameters in the electrical power distribution grid [20].

Voltage, kV	The Highest Transmitted Active Power, MW^2^	The Longest Transmission Distance, km
0.4	0.05–0.15	0.5–1
6–20	2–3	10–15
35	5–10	30–50
110	25–50	50–150

**Table 3:** Performance indicators of scientific research.

Scheme	Proposal Methodology	Power Transmission Line Events	Development Level
[27]	wide and deep CNN model to detect electricity theft in smart grids	electricity theft	complex
[28]	fault detection and isolation using IoT	failure and faults in electricity	simple, effective
[29]	Smart fault monitoring and normalizing of a power distribution system using IoT	failure and faults in SCADA	simple, effective
[30]	estimation for renewable energy integration using machine learning with the DLR method	solar radiation, wind speed, and ambient temperature	medium
[31]	optimal synchronization system for remotely located sensor	break and cause damage, issue with phase	complex
[32]	autonomously monitoring the current, voltage, oil level, and winding temperature of a distribution transformer using IoT	consumer-wise energy recording on current and voltage	simplex
[33]	cyber-attacks and coordinated cyber—physical attacks on power system	generation transformation and transmission, load current, and phase	very complex

**Table 4:** Error indicators of sensors in the remote monitoring device.

Testing Number	Error Voltage Sensor, %	Error Current Sensor, %	Error Temperature Sensor, %
1	0.99	0.12	0.7
2	1.23	0.74	0.66
3	0.15	1.01	0.45
4	0.4	0.8	1.001
5	0.49	0.69	0.2
6	1.37	−0.45	0.62
7	−0.89	0.68	0.35
8	1.16	−0.65	1.26
9	0.49	0.59	0.33
10	2.06	2.15	1.65
**Average** **error**	**0.74**	**0.56**	**0.72**

**Table 5:** Indicators of the probability of operation of lines for one month (720 h) without rejection.

Reliability Indicators	Different Times During the Month													
$t_{hours}$	48	96	144	200	240	288	300	360	400	480	528	600	672	720
$P_{{sys}_{1}}$	0.988	0.979	0.966	0.953	0.944	0.933	0.930	0.917	0.908	0.891	0.881	0.866	0.851	0.841
$P_{{sys}_{2}}$	0.989	0.980	0.969	0.957	0.949	0.939	0.936	0.924	0.916	0.9	0.890	0.876	0.862	0.853

**Table 6:** Indicators of the probability of the network operation without rejection for one year (8760 h).

**Reliability Indicators**	**Different Times During a Month**													
$t_{hours}$	48	96	144	200	240	288	300	360	400	480	528	600	672	720
$Model\;A-P_{{sys}_{1}}$	0.998	0.997	0.995	0.993	0.992	0.991	0.990	0.988	0.987	0.985	0.983	0.981	0.979	0.977
${Model\;B-P}_{{sys}_{2}}$	0.998	0.997	0.995	0.994	0.993	0.991	0.991	0.989	0.988	0.986	0.984	0.982	0.980	0.979
**Reliability Indicators**	**Different Times Three Months**													
$t_{hours}$	48	100	200	300	500	1000	1200	1400	1600	1800	2000	2040	2160	
$Model\;A-P_{{sys}_{1}}$	0.998	0.996	0.993	0.990	0.984	0.969	0.963	0.957	0.951	0.945	0.939	0.938	0.935	
${Model\;B-P}_{{sys}_{2}}$	0.998	0.997	0.994	0.991	0.985	0.971	0.965	0.960	0.954	0.949	0.943	0.942	0.939	
**Reliability indicators**	**Different Times During Six Months**													
$t_{hours}$	48	100	500	1000	2000	2500	3000	3480	3600	3720	3840	4080	4320	
$Model\;A-P_{{sys}_{1}}$	0.998	0.996	0.984	0.969	0.939	0.925	0.911	0.897	0.894	0.891	0.887	0.881	0.874	
${Model\;B-P}_{{sys}_{2}}$	0.998	0.997	0.985	0.974	0.943	0.930	0.916	0.903	0.900	0.897	0.894	0.888	0.882	
**Reliability indicators**	**Different Times During One Year**													
$t_{hours}$	48	100	500	1000	2000	3000	4000	5000	6000	7000	8160	8400	8760	
$Model\;A-P_{{sys}_{1}}$	0.998	0.996	0.984	0.969	0.939	0.911	0.883	0.856	0.830	0.804	0.776	0.770	0.762	
${Model\;B-P}_{{sys}_{2}}$	0.998	0.997	0.985	0.971	0.943	0.916	0.890	0.865	0.840	0.816	0.789	0.783	0.775	

**Table 7:** Comparative table of the proposed model with the results of other studies.

№	Researched Methods	Field of Application	Measurement Accuracy	Energy Efficiency	Reliability Indicators
Method 1	expert assessment based on analytical data	monitoring system of heavy engineering	0.95	0.87	0.89
Method 2	fusion method of three-state reliability evaluation	restorable monitoring system	0.96	0.88	0.97
Method 3	automatic generation of a fault tree evaluation method	automated industrial applications	0.91	0.9	0.91
Method 4	proposal method	remote monitoring system of low voltage overhead power line	0.97	0.95	0.98

## Data Availability

The original contributions presented in the study are included in the article, further inquiries can be directed to the corresponding authors.
